# Editorial: Innovative tumor therapies: targeting angiogenesis and metabolism

**DOI:** 10.3389/fcell.2025.1639613

**Published:** 2025-07-10

**Authors:** Kaiying Yang, Yi Ji

**Affiliations:** ^1^ Department of Pediatric Surgery, Guangzhou Women and Children’s Medical Center, National Children’s Medical Center for South Central Region, Guangzhou Medical University, Guangzhou, China; ^2^ Department of Pediatric Surgery, West China Hospital, Sichuan University, Chengdu, China

**Keywords:** angiogenesis, metabolic reprograming, hypoxia, vascular tumor, glycolysis

## Introduction

Angiogenesis and metabolic reprogramming are two key hallmarks of tumors. Angiogenesis, the formation of new blood vessels, supplies essential nutrients and oxygen to tumors, facilitating their growth and metastasis. Concurrently, tumor cells undergo metabolic reprogramming to adapt to the tumor microenvironment and meet their increased energy demands. These processes play critical roles in the proliferation and progression of benign tumors (such as infantile hemangioma, IH), borderline tumors (such as Kaposiform hemangioendothelioma, KHE), and malignant tumors. Therefore, targeting angiogenesis and cellular metabolism has emerged as a promising and essential strategy in tumor therapy. This Research Topic focuses on overcoming the challenges of targeting tumor angiogenesis and metabolism by elucidating their molecular crosstalk, uncovering new therapeutic targets, and assessing the clinical promise of combination strategies. Seven articles were included in this Research Topic, comprising five review articles and two original research papers.

## Metabolic and angiogenic features of IH and KHE

As the most common benign vascular tumor in children, IH develops primarily through pathological angiogenesis (Xiang et al., 2024). Compared with HUVECs, hemangioma-derived endothelial cells (HemECs) exhibit significantly higher glycolytic activity and elevated expression of glycolytic key enzymes, indicating that IH is a highly glycolytic tumor and primarily relies on glycolysis for energy supply (Yang et al., 2023a). Targeted inhibition of the glycolytic regulator PFKFB3 markedly reduces HemECs proliferation, migration, tube formation *in vitro*, and tumorigenesis *in vivo* in nude mice (Yang et al., 2023b). KHE is a locally aggressive, borderline vascular tumor, with Kasabach-Merritt phenomenon occurring in approximately 70% of cases and significantly increasing mortality. Dysregulated angiogenesis and lymphangiogenesis are central to its pathogenesis (Ji et al., 2020). Currently, sirolimus is emerging as the first-line treatment for KHE (Zhou et al., 2025), and one of its key therapeutic mechanisms involves the inhibition of the PI3K/Akt/mTOR pathway, thereby suppressing angiogenesis (Qiu et al., 2025).

## Coordinating role of hypoxia in tumor angiogenesis and metabolism

Hypoxia, another hallmark of tumors, activates the expression of HIF-1α, which subsequently upregulates multiple target genes involved in angiogenesis and cellular metabolism. Liu et al. reported that elevated HIF-1α expression in proliferating IH induces downstream pro-angiogenic factors such as VEGF, enhancing HemECs proliferation and migration. Similarly, HIF-1α is highly expressed in KHE, where it upregulates VEGFC and VEGFR3 to promote angiogenesis. Sirolimus partly exerts its therapeutic effect by suppressing this pathway, downregulating HIF-1α and its downstream targets like VEGF, thereby inhibiting angiogenesis. Furthermore, their study provides additional insights into how hypoxia regulates angiogenesis and metabolic reprogramming. During angiogenesis, endothelial cells (ECs) must transition from quiescence to proliferation state, a shift that requires substantial energy for their migration and proliferation. Glycolysis is the predominant metabolic pathway in ECs, as it generates over 85% of their total ATP. HIF-1α directly upregulates the expression of glucose transporter such as GLUT1 and key glycolytic enzymes including PFKFB3, PKM2 and LDHA, enhancing glycolytic activity in vascular tumor such as IH. Elevated glycolysis provides ECs with biosynthetic precursors, ATP, enhanced hypoxia tolerance, and reduced ROS production, thereby facilitating angiogenesis under hypoxic conditions (Falkenberg et al., 2019). This highlights the interplay between angiogenesis and metabolic reprogramming and the potential for targeted therapies.

## Hypoxia-induced limitations and advances in photodynamic therapy

In addition to promoting angiogenesis and metabolic reprogramming, hypoxia also impairs the efficacy of photodynamic therapy (PDT), an oxygen-dependent treatment widely applied to tumors such as IH due to its minimal invasiveness, spatiotemporal precision, and high biocompatibility. Mechanistically, PDT relies on photosensitizers activated by light to convert intracellular oxygen into cytotoxic ROS, resulting in oxidative damage and subsequent cell death. Recognizing hypoxia as a limiting factor, Lv et al. reviewed recent advances in photodynamic nanotechnological strategies that aim to enhance the efficacy of PDT in tumor treatment. Their study elucidated the mechanisms by which targeting angiogenesis or cellular metabolism can further potentiate PDT efficacy. This research offers valuable insights and potential strategies to overcome current challenges associated with PDT, thereby advancing its clinical utility in cancer treatment.

## Metabolic reprogramming and angiogenesis across pediatric and gastrointestinal tumors

Building upon these insights into hypoxia-driven angiogenesis and metabolic modulation, subsequent studies further illustrate the mechanistic and therapeutic potential of targeting these processes in a broader range of tumors. Hepatoblastoma (HB) is the most common malignant liver tumor in children. The combination of anti-angiogenic agents with chemotherapy has been shown to significantly enhance treatment efficacy in HB, underscoring the critical role of angiogenesis in its pathogenesis. Kong et al. provided a comprehensive overview of metabolic reprogramming in HB and its regulation of angiogenesis via several key signaling pathways, including the Wnt/β-catenin, VEGF, and PI3K/Akt pathways. Lv et al. provided insights into the therapeutic implications of cellular metabolic pathways, including glycolysis, oxidative phosphorylation, amino acid metabolism, and lipid metabolism, in pediatric thoracic tumors. Li et al. summarized the molecular mechanisms of key angiogenic regulators, including VEGF, HIFs, and non-coding RNAs, in gastrointestinal tumors. They also emphasized targeting angiogenesis through anti-angiogenic agents and vascular normalization as a therapeutic strategy.

## Challenges in anti-angiogenic therapy and emerging metabolism-targeted strategies

Although targeting angiogenesis is a critical strategy in cancer therapy, its clinical efficacy is often limited by drug resistance, tumor recurrence, and severe adverse events. One key mechanism underlying these shortcomings is the compensatory upregulation of alternative pro-angiogenic factors when a single angiogenic factor or receptor is inhibited, thereby restoring angiogenesis and reducing therapeutic effectiveness (Li et al., 2019). Yang et al. reported a marked increased risk of gingival and tumor hemorrhage with angiogenesis inhibitors, especially in females, highlighting potential safety concerns of monotherapy. These limitations underscore the urgent need for alternative or adjunctive therapeutic strategies. To overcome these challenges, targeting the angiogenic engine—EC metabolism, a center driving force of angiogenesis—rather than angiogenic drivers such as growth factors or their receptors, can effectively suppress angiogenesis regardless of the number pro-angiogenic signals (Li et al., 2019; You et al., 2023; Tufail et al., 2024). Moreover, integrating metabolic therapies with conventional anti-angiogenic agents may help mitigate resistance and toxicity while enhancing therapeutic efficacy, offering a more comprehensive and promising direction for future cancer treatment.

## Conclusion

The articles in this Research Topic collectively underscore the mechanistic interdependence between angiogenesis and metabolic reprogramming as a center drive of tumor development and therapy. Rather than offering isolated summaries, these studies together demonstrate that targeting the angiogenesis–metabolic axis holds therapeutic potential across diverse tumor types. However, the spatiotemporal regulation of this axis remains incompletely understood, particularly across distinct tumor microenvironments and disease stages. Future studies should clarify its heterogeneity and guide rational combination therapies.
